# Supplementary material for: Superconductivity in an extreme strange metal
Source: Nat Commun. 2021 Jul 21;12:4341. doi: 10.1038/s41467-021-24670-z (PMC8295387; doi:10.1038/s41467-021-24670-z)
Supplement: Supplementary file 1 — Supplementary Information [file 41467_2021_24670_MOESM1_ESM.pdf]

Supplementary Information

Superconductivity in an extreme strange metal

D. H. Nguyen,[1] A. Sidorenko,[1] M. Taupin,[1] G. Knebel,[2] G. Lapertot,[2]
E. Schuberth,[3] and S. Paschen[1,*]

[1]Institute of Solid State Physics, Vienna University of Technology, 1040 Vienna, Austria
[2]Université Grenoble Alpes, CEA, IRIG, PHELIQS, 38000 Grenoble, France
[3]Technische Universität München, 85748 Garching, Germany

Supplementary Note 1: Analysis of resistivity vs magnetic field isotherms

In ^{174}YbRh$_2$Si$_2$ the electrical resistivity vs magnetic field isotherms (Fig. 3b) at the lowest temperatures show clear signs of reentrance. To extract meaningful upper critical field B_{c2} and critical temperature T_c values is thus more involved than in the case of a single superconductor–normal conductor phase boundary (where the criterion of a 50% resistivity drop is frequently adopted). We have thus devised a simple 'multi-transition model' to separate the different effects. A single magnetic field-driven transition from a superconductor to a normal conductor is (phenomenologically) described by

$$\rho/\rho_n = \frac{1}{1+e^{-k(B-B_{c2})}} \equiv \mathscr{F}(B) \tag{1}$$

where B_{c2} represents the half-hight (50% resistivity drop) criterion and k is a measure of the sharpness of the transition. The effect of first entering and then leaving a superconducting phase II as function of magnetic field is then captured by

$$\rho/\rho_n = 1 - (1-\mathscr{F}_{\text{left}}) \cdot (1-\mathscr{F}_{\text{right}}) = \mathscr{F}_{\text{II}}(B)\ , \tag{2}$$

and the total effect of starting in a superconducting phase I, leaving it, and passing through the superconducting phase II by

$$\rho/\rho_n = \mathscr{F}_{\text{I}}(B) \cdot \mathscr{F}_{\text{II}}(B)\ , \tag{3}$$

as shown in Supplementary Fig. 1. This corresponds to a parallel circuit. Note that the total trace (black curve) does not cross the 50% line at the same fields as the separate traces (blue, green, and red lines). Not using such a model will wash out the transitions between adjacent superconducting phases.

In fitting the $\rho(B)$ isotherms of ^{174}YbRh$_2$Si$_2$ (Fig. 3b), only for the lowest temperature (0.6 mK) the fit fully converged with all fit parameters (k_i, $B_{\rm c2,i}$, i = I, II-left, II-right) open. Thus, to enable fits also at higher temperatures, we used the $B_{\rm c2,I}$ value determined in this lowest-temperature fit and the critical temperature in zero field to approximate the phase boundary with the mean-field form $B_{\rm c2}(T) = B_{\rm c2}(T=0) \cdot [1-(T/T_{\rm c})^2]$, and fixed $B_{\rm c2,I}$ in the higher-temperature fits to the corresponding values. All other parameters were left open and the fits converged.

Also the low-temperature $\rho(B)$ isotherms of YbRh$_2$Si$_2$ (Fig. 3a) show 'double transition' signatures, but because they are less pronounced than in ^{174}YbRh$_2$Si$_2$ we have refrained from doing a similar analysis there (the critical fields are simply determined by the 50% resistivity drop criterion, as explained in the main text).

Supplementary Note 2: Estimates on Planckian dissipation

In a material with Planckian dissipation, a linear-in-temperature electrical resistivity arises when the scattering rate $1/\tau$ reaches the Planckian limit, $k_{\rm B}T/\hbar$. To test whether this is the case in YbRh$_2$Si$_2$, we have determined the proportionality coefficient α in

$$\frac{1}{\tau} = \alpha \frac{k_{\rm B}T}{\hbar} \quad (4)$$

in two different ways. In a first approach, we use the simple Drude form

$$\rho = \frac{m}{ne^2}\frac{1}{\tau} \quad (5)$$

for the inelastic part

$$\rho_{\rm in} = A'T \quad (6)$$

of the strange-metal electrical resistivity $\rho = \rho_0 + \rho_{\rm in}$, identify $1/\tau$ in Supplementary Eq. 5 with Supplementary Eq. 4, and obtain

$$\alpha = \frac{ne^2}{m}\frac{\hbar}{k_{\rm B}}A' \,. \quad (7)$$

Here n is the charge carrier concentration and m the charge carriers' effective mass. Because YbRh$_2$Si$_2$ is a multiband conductor[41], estimating n from Hall effect measurements[27,28] in a simple one-band model may introduce a sizable error. Thus, instead, we determined n from the Ginzburg-Landau coherence length $\xi_{\rm GL}$, the superconducting transition temperature $T_{\rm c}$, and the Sommerfeld coefficient γ, via the relation

$$n = \left(\frac{\xi_{\rm GL}T_{\rm c}\gamma}{7.95\cdot 10^{-17}}\right)^{3/2} \quad \text{in cgs units,} \quad (8)$$

as described in refs. 42,43. $\xi_{\rm GL}$ and $T_{\rm c}$ are taken from Table II. γ is calculated from the zero-field A coefficient of the Fermi liquid form $\rho = \rho_0 + AT^2$ (from Table I) via the universal Kadowaki-Woods ratio $A/\gamma^2 = 10^{-5}\,\mu\Omega{\rm cm}({\rm molK})^2/({\rm mJ})^2$. We expect this value, $\gamma_{\rm KW}^{0\,\rm T}$ (see Table I) to be a more reliable estimate of the Sommerfeld coefficient than the directly measured specific heat data because the phase transition anomaly from the Néel transition[16,17] is superimposed on the data and difficult to subtract. The corresponding averaged Fermi wavevector

$$k_{\rm F} = (3\pi^2 n)^{1/3} \tag{9}$$

is given in Table II for both $YbRh_2Si_2$ and $^{174}YbRh_2Si_2$. This $k_{\rm F}$ and $\gamma_{\rm KW}^{0\,\rm T}$ are used to calculate the effective mass as

$$m = \frac{3\hbar^2}{k_{\rm B}^2}\frac{\gamma_{\rm KW}^{0\,\rm T}}{k_{\rm F}}\ . \tag{10}$$

The α values thus obtained from Supplementary Eq. 7 are 0.011 and 0.0065 for $YbRh_2Si_2$ and $^{174}YbRh_2Si_2$, respectively (Table II), much smaller than $\alpha \approx 1$ expected for Planckian dissipation.

Our second approach makes use of recent optical conductivity measurements on $YbRh_2Si_2$[19], which showed that the inverse of the real part of the inelastic optical conductivity (an optical resistivity) is linear in frequency ν:

$$\frac{1}{{\rm Re}(\sigma_{\rm in})} = A''\nu\ . \tag{11}$$

By equating Supplementary Eq. 6 and Supplementary Eq. 11 and using Supplementary Eq. 4 for $\nu = 1/\tau$ we can calculate α as

$$\alpha = \frac{\hbar}{k_{\rm B}}\frac{A'}{A''}\ , \tag{12}$$

which yields 0.0062, in good agreement with the estimates from approach 1.

Supplementary Figures

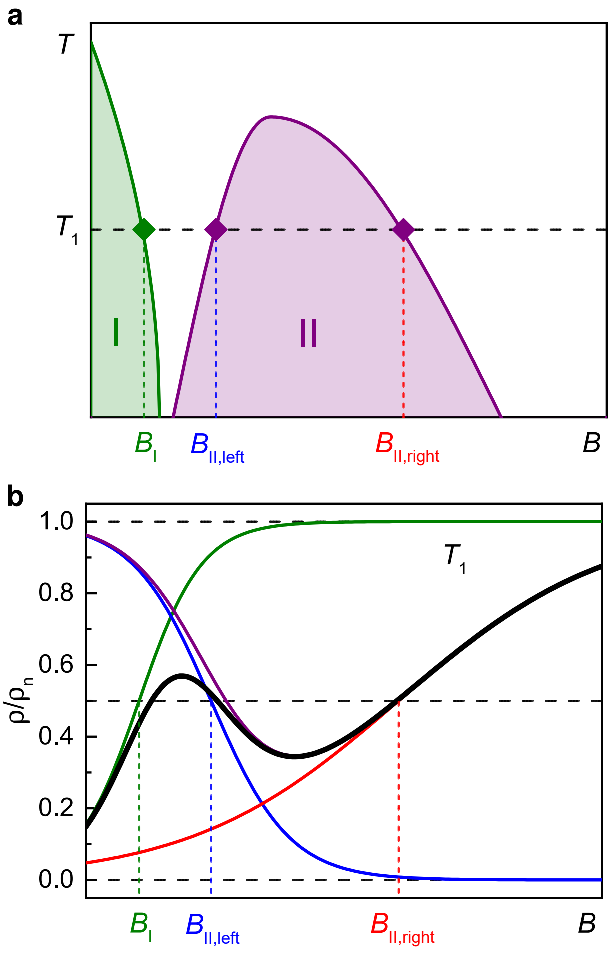

Supplementary Fig. 1: Determination of critical fields in a conductor with two adjacent superconducting phases. **a** Example of two superconducting phases, I and II, described by our simple 'multi-transition model' (see text). **b** Electrical resistivity ρ, normalized to its value in the normal state ρ_{n} just above the onset of superconductivity, as function of the applied magnetic field B, at a fixed temperature T_1. The total $\rho/\rho_{\mathrm{n}}(B)$ trace (black) is compose of three logistic functions (green, blue, red), with transitions at B_{I}, $B_{\mathrm{II,left}}$, and $B_{\mathrm{II,right}}$, as described in the text.

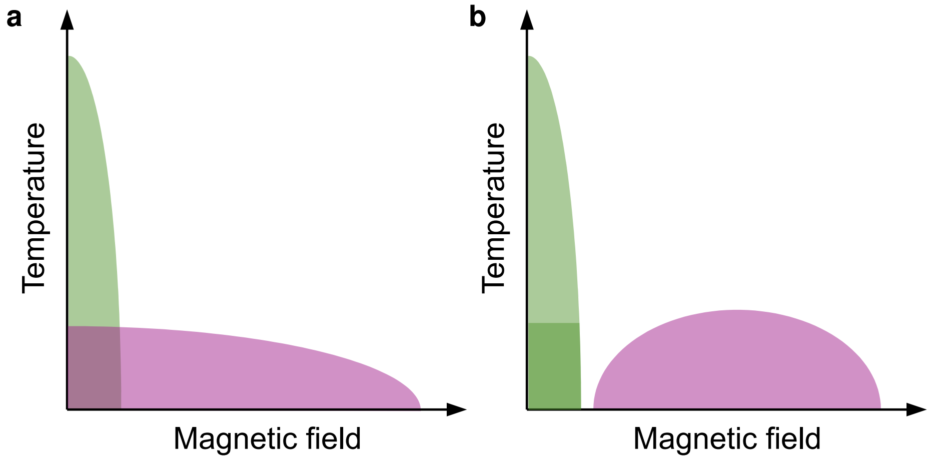

Supplementary Fig. 2: Cartoons of possible superconducting phases in $YbRh_2Si_2$. a Two intersecting phases. **b** Two separated phases, with an internal phase boundary in the low-field phase. Both are consistent with the combined results from the present work and ref. 33.

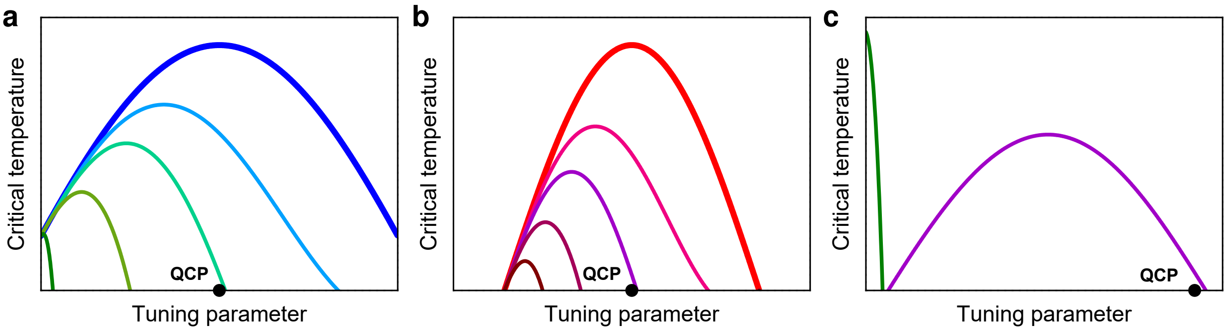

Supplementary Fig. 3: Cartoons of magnetic-field effect on superconducting domes around a quantum critical point (QCP). a The fat blue curve is a hypothetical $T_c(B)$ curve where any (hostile) effect of the magnetic field on superconductivity (from Pauli or orbital limiting) is imagined to be absent. For the successive curves (from blue to green), an increasingly strong field effect is considered (using a simple mean-field-type suppression of T_c by the magnetic field B, which increases from zero along the tuning parameter axis). **b** Same as **a**, but for a superconducting phase with a dome that does not extend to the zero of the tuning parameter axis (fat red curve). Again, an increasingly strong field effect is added for the successive curves (from red to brown). **c** Lowest curve from panel **a** (green) and middle curve of panel **b** (purple), scaled in absolute values. A material with two QCP-derived superconducting phases, one with stronger pairing but larger field sensitivity (as in panel **a**, e.g., a spin-singlet superconductor) and one with weaker pairing but also weaker field sensitivity (as in panel **b**, e.g., a spin-triplet superconductor), could display such a magnetic-field-tuned phase diagram.

Supplementary References

41. Friedemann, S., Wirth, S., Oeschler, N., Krellner, C., Geibel, C., Steglich, F., MaQuilon, S., Fisk, S., Paschen, S. & Zwicknagl, G. Hall effect measurements and electronic structure calculations on $YbRh_2Si_2$ and its reference compounds $LuRh_2Si_2$ and $YbIr_2Si_2$. *Phys. Rev. B* **82**, 035103 (2010).

42. Rauchschwalbe, U., Lieke, W., Bredl, C. D., Steglich, F., Aarts, J., Martini, K. M. & Mota, A. C. Critical fields of the "heavy-fermion" superconductor $CeCu_2Si_2$. *Phys. Rev. Lett.* **49**, 1448–1451 (1982).

43. Orlando, T. P., McNiff, Jr., E. J., Foner, S. & Beasleya, M. R. Critical fields, Pauli paramagnetic limiting, and material parameters of Nb_3Sn and V_3Si. *Phys. Rev. B* **19**, 4545 (1979).
